# Motivators and barriers for studying podiatry in Australia and New Zealand: A mixed methods study

**DOI:** 10.1002/jfa2.70004

**Published:** 2024-09-04

**Authors:** Michelle R. Kaminski, Glen A. Whittaker, Caroline Robinson, Matthew Cotchett, Malia Ho, Shannon E. Munteanu, Mollie Dollinger, Sia Kazantzis, Xia Li, Ryan S. Causby, Mike Frecklington, Steven Walmsley, Vivienne Chuter, Sarah L. Casey, Burke Hugo, Daniel R. Bonanno

**Affiliations:** ^1^ Discipline of Podiatry School of Allied Health Human Services and Sport La Trobe University Melbourne Victoria Australia; ^2^ Department of Podiatry Monash Health Melbourne Victoria Australia; ^3^ School of Primary and Allied Health Care Monash University Melbourne Victoria Australia; ^4^ School of Allied Health, Exercise and Sports Sciences Charles Sturt University Albury New South Wales Australia; ^5^ Faculty of Health Sciences Curtin University Bentley Western Australia Australia; ^6^ Department of Physiotherapy Melbourne School of Health Sciences The University of Melbourne Melbourne Victoria Australia; ^7^ Mathematics and Statistics School of Computing, Engineering and Mathematical Science La Trobe University Melbourne Victoria Australia; ^8^ Allied Health and Human Performance Unit University of South Australia Adelaide South Australia Australia; ^9^ Department of Podiatry School of Clinical Sciences Faculty of Health and Environmental Sciences Auckland University of Technology Auckland New Zealand; ^10^ Discipline of Podiatry School of Clinical Sciences Faculty of Health Queensland University of Technology Brisbane Queensland Australia; ^11^ School of Health Science Western Sydney University Campbelltown New South Wales Australia; ^12^ Discipline of Podiatry School of Health Sciences Western Sydney University Campbelltown New South Wales Australia; ^13^ Division of Podiatric Medicine and Surgery School of Allied Health University of Western Australia Perth Western Australia Australia

**Keywords:** career choice, health workforce, podiatry, students, university

## Abstract

**Background:**

Podiatry enrolments at Australian and New Zealand universities have decreased by 17.3% since 2015, which threatens the profession's sustainability and the health and wellbeing of Australian and New Zealand people and communities. Reasons for this decline remain unclear due to insufficient evidence on factors influencing career choices. The overarching aim of this study was to identify motivators and barriers for studying podiatry in Australia and New Zealand.

**Methods:**

This study used a convergent mixed methods design. Students enrolled in (i) podiatry and (ii) relevant non‐podiatry health, sport or science programs at nine Australian and one New Zealand university, were invited to participate in an online survey. First‐year podiatry students were also invited to participate in an online workshop. Quantitative data were analysed using descriptive statistics and linear/logistic regression models. Three independent assessors used inductive thematic analysis for the qualitative data.

**Results:**

Overall, 278 *podiatry* students (mean age 24.9 ± 8.5 years, 65.1% female) and 553 *non‐podiatry* students (mean age 24.8 ± 8.2 years, 75.4% female; 32.2% from physiotherapy and 29.1% from occupational therapy) responded to the survey. Interest in a health‐related career, wanting to make a difference to people's health, and opportunity to care for people from different backgrounds/age groups were key motivating factors among podiatry students. Barriers to studying podiatry were encountered by 28.1% of podiatry students. Thematic analysis identified seven themes concerning career choice, which are as follows: (i) awareness of profession and scope of practice; (ii) stereotypes and negative perceptions of the profession; (iii) awareness of career pathways; (iv) job prospects and earning potential; (v) working with people and building relationships; (vi) podiatry is not the first preference; and (vii) barriers which limit student enrolment.

**Conclusions:**

There are a variety of factors that motivate and influence students to study podiatry, however, altruistic reasons are most highly rated. Allied health students have limited understanding of the scope of practice and career opportunities in podiatry. Additionally, the podiatry profession often faces negative stereotypes. Further work is required to reverse the negative stereotypes and perceptions of podiatry and build knowledge of the profession's scope of practice, career pathways/opportunities, job prospects and earning potential.

**Abbreviations**
AUT: Auckland University of Technology
CI: Confidence interval
COREQ: Consolidated criteria for reporting qualitative research
CQU: Central Queensland University
CSU: Charles Sturt University
F: Female
GRAMMS: Good reporting of a mixed methods study
IQR: Interquartile range
LTU: La Trobe University
M: Male
NSW: New South Wales
NZ: New Zealand
OR: Odds ratio
QLD: Queensland
SA: South Australia
SCU: Southern Cross University
SD: Standard deviation
UK: United Kingdom
UniSA: University of South Australia
UON: University of Newcastle
UWA: University of Western Australia
VIC: Victoria
WA: Western Australia
WSU: Western Sydney University

## 1 BACKGROUND

Allied health represents the largest clinical workforce within primary healthcare in Australia [1, 2], which is similar to that of Aotearoa (New Zealand) [3]. Podiatry is an allied health profession that specialises in the ‘prevention, diagnosis, treatment, and rehabilitation of medical and surgical conditions of the feet and lower limbs’ [4, 5, 6]. Podiatrists work within a variety of clinical settings where service provision and care co‐ordination are often shared with other health practitioners, such as acute inpatient services, rehabilitation, community health, aged care and private practice [6]. The podiatry profession has a relatively small occupation share of employment in Australia and New Zealand, with 5942 registered general podiatrists and 40 registered podiatric surgeons in Australia (data as of December 2023) [7], and 472 registered general podiatrists and two registered podiatric surgeons in New Zealand (data as of March 2023) [8]. Australia has 22.4 practicing podiatrists for every 100,000 people (data as of June 2022) [9], while New Zealand has 9.2 practicing podiatrists per 100,000 people (data as of March 2023) [8].

The increasing demand for podiatry services in Australia and New Zealand, relative to workforce size, is affecting service sustainability and limiting the capacity of podiatry health services to meet diverse clinical needs [5, 9, 10, 11]. Key drivers of podiatry demand include ageing populations, increasing prevalence of chronic disease, service awareness and accessibility, funding arrangements, referral practices and higher community and clinician expectations regarding provision of podiatry care [9, 10, 11, 12]. Despite this growing demand, undergraduate podiatry enrolments across Australian and New Zealand university programs have experienced an overall 17.3% decline since 2015 [13]. There are significant consequences related to the current decline in student enrolments, including (i) threats to the viability of podiatry programs, (ii) workforce shortages, (iii) reduced capacity of podiatrists to work to their full scope of practice (i.e., “the professional activities that a practitioner is educated, competent and authorised to perform, and for which they are accountable” (p.11)) [14] due to increased workloads from fewer graduates entering the workforce [15], (iv) threats to the profession's growth and reduced impact of podiatry advocacy at government‐level, (v) burnout in existing podiatry workforce [16] and (vi) negative impact on the health and wellbeing of the Australian and New Zealand populations. For example, workforce shortages and increased workloads due to fewer graduates may lead to gaps in podiatry services, particularly in rural and remote areas, increasing health inequities and reducing access to healthcare for those with the greatest need [17, 18, 19].

Understanding why people choose to study podiatry will provide valuable insight to address declining podiatry enrolments. Recent national survey data from England reported altruistic motivators for choosing to study podiatry, such as an ‘intellectually‐stimulating and challenging role’ [20]. Similarly, a recent qualitative study [21] from the United Kingdom (UK) identified factors that attract Generation Z (people born between 1995 and 2010) to a career in podiatry, including: accessibility as a course and career; attractive career status; and broad scope of practice [21].

There is a lack of current data relevant to the Australian and New Zealand contexts. One study [22] completed in 2003, reported that podiatry students rate intrinsic rewards (e.g., to help other people) higher than extrinsic rewards (e.g., to earn a good income) when choosing this career path; however, it is unknown if these findings are representative of current students. It is also unclear how the interplay between personal and professional factors influences career pathway decisions for podiatry students, and how this differs to other health, sport or science students.

Therefore, this study aimed to identify the motivators and barriers for studying podiatry among current students enrolled in podiatry programs across Australia and New Zealand. The secondary aim of this research was to identify factors influencing students to study other relevant health, sport or science (non‐podiatry) programs.

## 2 METHODS

### 2.1 Ethics approval

This study was approved by the La Trobe University (LTU) Human Research and Ethics Committee (HEC21057) and ethics approval was also obtained from the following institutions via mutual acceptance applications: Auckland University of Technology (AUT) (21/161), Central Queensland University (CQU) (22978), Charles Sturt University (CSU) (H21077), Southern Cross University (SCU) (2021/043), University of Newcastle (UON) (H‐2021‐0276), University of South Australia (UniSA) (203889), University of Western Australia (UWA) (2021/ET000372) and Western Sydney University (WSU) (H14404). All participants provided informed consent prior to data collection.

### 2.2 Study design and reporting

This study used a convergent mixed methods design, where quantitative and qualitative data were collected and analysed in parallel and the approach to integration involved merging [23, 24]. The study is reported in accordance with the consolidated criteria for reporting qualitative research (COREQ) framework and the Good Reporting of A Mixed Methods Study (GRAMMS) checklist [25, 26]. See Additional File 1 for the completed GRAMMS checklist.

### 2.3 Research team

Our team included researchers with experience in quantitative and qualitative research investigating foot and ankle pathology, chronic disease, health promotion and culturally responsive practice. All workshops were facilitated by a Lecturer in Student Success (MD) with 5 years of experience in higher education and co‐facilitated by a Senior Lecturer in Podiatry (DRB) with 16 years of experience in higher education, and three Podiatry Program Leads (CR, MH, RSC) with 39, 5 and 20 years of experience in higher education, respectively. A research assistant (SK) with 11 years of experience in clinical podiatry also assisted in facilitating the workshops. Thematic analysis was conducted by two Podiatry Program Leads (CR and MH) and a Senior Lecturer (MC) with 16 years of experience in higher education.

### 2.4 Participants

#### 2.4.1 Online survey

All currently enrolled podiatry students, and students enrolled at the same institution in relevant ‘non‐podiatry’ programs in Australia and New Zealand, were invited to participate in an online survey. For this study, the ‘non‐podiatry’ programs included nursing, physiotherapy, exercise science, occupational therapy, speech pathology, dietetics/nutrition, orthoptics, prosthetics and orthotics and various science programs (e.g., health science, biomedicine). Students within these programs hereinafter are referred to as ‘non‐podiatry students’.

#### 2.4.2 Workshops

All first‐year podiatry students enrolled at AUT, CSU, LTU and UniSA were invited to participate in one of four workshops. These four institutions were selected for the following reasons: (i) LTU and UniSA both have relatively large student cohorts (combined enrolments of ∼390 students across four year levels) with differing geographies and contrasting concerns regarding student demand (i.e., LTU enrolments have decreased since 2015 while UniSA enrolments have, until recently, remained stable); (ii) LTU and UniSA are metropolitan institutions and CSU has a regional location; and (iii) LTU, UniSA and CSU are located in Australia and AUT is in New Zealand. First‐year students were chosen for the qualitative component of this study as they are likely to have a more accurate recall of the reasons they chose to study podiatry, given their recent decision to enter the program. Additionally, their perspectives are less likely to be influenced by their experiences within the course over several years, providing a clearer insight into initial perceptions and motivations.

### 2.5 Sample size

An a priori sample size calculation for the podiatry student survey component of this study estimated that 264 participants were required, based on the following: (i) 880 podiatry students across all universities, (ii) expected response rate of ∼30%, (iii) a confidence level of 95% and (iv) a margin of error of 5% [27].

### 2.6 Recruitment

Podiatry Program Leads from 10 university podiatry programs across Australia and New Zealand^1^ were initially contacted via email and invited to participate in this study. Of these 10 institutions, only one university declined to participate. Once the participating universities were established, Program Leads from non‐podiatry programs were also contacted via email and invited to participate.

### 2.7 Data collection

#### 2.7.1 Online survey

The online survey tool (REDCap®, Research Electronic Data Capture, Vanderbilt, University) for the podiatry and non‐podiatry students consisted of open and closed questions (Additional Files 2 and 3). Both surveys included questions on demographics and the motivators, influencers and barriers for the students' respective career choices. The podiatry survey also included questions on marketing of the profession, while the non‐podiatry survey included questions on knowledge and perceptions of the podiatry profession. The podiatry survey component was initially piloted on first‐year podiatry students (*n* = 30; response rate of 29.7%) at three Australian universities offering podiatry programs: LTU (HEC20415), UniSA (203555) and CSU (H20344). The survey content for the current study was adapted based on feedback obtained from the pilot study.

Students received an email invitation from a university staff member (e.g., year level coordinator) with a link to the survey. To maximise response rates, reminder emails (three in total) were sent to students weekly over a 4‐week data collection period. Overall, the online survey was available between March 7, 2022 and April 14, 2022.

#### 2.7.2 Workshops

All workshops were conducted over an 8‐day period, commencing in March 2022. Each 60–90‐min workshop was conducted via Zoom videoconferencing (San Jose, California, USA) [28], facilitated by a Lecturer in Student Success (MD) using a co‐design workshop approach called ‘CoLabs’ [29], and co‐facilitated by members of the research team (DRB, CR, MH, RSC and SK). The workshops comprised interactive semi‐structured interviews using open‐ and close‐ended questions, and a range of activities to promote student reflection and feedback [29]. The first activity required students to create a mind map using Jamboard^TM^ electronic whiteboard (Google Inc.). Students were asked to write down what they thought of when they heard the word ‘podiatry’. The second activity required students to contribute to an online discussion forum using Padlet^®^ software. Students were asked to reflect on several questions relating to personal motivators and barriers for studying podiatry, and to share their insights into marketing of podiatry programs and the podiatry profession. The third activity required students to collaborate in small groups to co‐create a ‘campaign’ intended to attract more students to podiatry. The campaign instructions included considerations for key selling points relevant to prospective students, dissemination and/or roll‐out of the campaign, and the unique features of the podiatry profession. All data obtained from the workshop activities were collated in NVivo^®^ (Lumivero).

### 2.8 Data analysis

#### 2.8.1 Quantitative data

Descriptive statistics were used to report participant characteristics and data obtained from the closed survey questions. Data were expressed as mean (standard deviation, SD) or median (interquartile range, IQR). The Mann–Whitney *U* test was used for between‐group comparisons of the ordinal data. Associations between personal and professional factors that influence career pathway decisions for podiatry and non‐podiatry students were explored using linear and/or logistic regression (depending on data type). All statistical analyses were performed using R (version 4.3.2, R Foundation for Statistical Computing). Statistical significance was set at the two‐sided conventional level of *p* ≤ 0.05.

#### 2.8.2 Qualitative data

Responses to open questions in the online survey from podiatry (*n* = 278) and non‐podiatry (*n* = 553) students, and qualitative data collected in the podiatry student workshops (*n* = 9) were used to generate themes in this study. All qualitative data were initially collated in a Microsoft Excel^®^ spreadsheet (Redmond) and were then analysed using inductive thematic analysis by three independent assessors (CR, MH, MC). Coding was completed using NVivo^®^ software (Lumivero). Final themes were agreed by consensus. Thematic analysis was conducted as per the six phases outlined by Braun and Clarke [30]: (i) data familiarisation; (ii) initial coding; (iii) theme searching; (iv) reviewing themes; (v) defining and naming themes; and (vi) manuscript preparation [30].

#### 2.8.3 Data integration

Using a convergent mixed methods design, the analysis and interpretation of the quantitative and qualitative data were conducted separately by researchers MRK, GAW, DRB, CR, MC and MH, followed by integration of the results derived from each of the analyses by researchers MRK, CR, MC and MH. The aim was to arrange and integrate these results and findings systematically, forming a cohesive narrative or argument [23, 24].

## 3 RESULTS

### 3.1 Participant characteristics

A total of 278 podiatry students and 553 non‐podiatry students participated in the online surveys, with good representation across all year levels. Table 1 and Additional File 4 outline the participant characteristics. The non‐podiatry student surveys had representation from a wide range of disciplines including physiotherapy (32.2%), occupational therapy (29.1%), science (7.1%) orthoptics (6.7%), sport and exercise science (6.3%), nursing (5.4%), prosthetics and orthotics (4.7%), paramedicine (3.3%), psychology (2.2%), speech pathology (1.1%), oral health (1.1%), exercise physiology (0.7%) and other programs (0.2%). Overall, most of the podiatry and non‐podiatry students were enrolled full‐time (92.4% and 85.9%, respectively), and a large proportion of students had prior qualifications (45.7% and 39.4%, respectively).

**TABLE 1:** Participant characteristics.

	Podiatry (*n* = 278)	Non‐podiatry (*n* = 553)
Age, *mean (SD)*	24.9 (8.5)	24.8 (8.2)
Female sex, *n (%)*	181 (65.1)	417 (75.4)
Carer responsibilities, *n (%)*	37 (13.3)	91 (16.5)
Prior educational qualifications, *n (%)* ^a^	127 (45.7)	218 (39.4)
Certificate I, II, III and IV	64 (23.1)	157 (28.4)
Diploma, advanced diploma and associate degree	21 (7.6)	89 (16.1)
Bachelor degree	60 (21.6)	76 (13.7)
Master's degree	9 (3.2)	9 (1.6)
Doctoral degree	1 (0.4)	2 (0.4)
University, *n (%)* ^a^		
Auckland University of Technology	15 (5.4)	88 (15.9)
Central Queensland University	11 (4.0)	45 (8.1)
Charles Sturt University	19 (6.8)	126 (22.8)
La Trobe University	48 (17.3)	66 (11.9)
Southern Cross University	8 (2.9)	0 (0)
The University of Newcastle	40 (14.4)	62 (11.2)
University of South Australia	75 (27.0)	90 (16.3)
University of Western Australia	19 (6.8)	8 (1.4)
Western Sydney University	43 (15.5)	67 (12.1)
Study load, *n (%)*		
Part‐time^b^	21 (7.6)	78 (14.1)
Full‐time	257 (92.4)	475 (85.9)
Year of study, *n (%)* ^a^		
First	76 (27.3)	152 (27.5)
Second	68 (24.5)	152 (27.5)
Third	81 (29.1)	152 (27.5)
Fourth	53 (19.1)	96 (17.4)
International student, *n (%)* ^a^	17 (6.1)	19 (3.4)
Primary role in year prior to commencing program of study, *n (%)* ^a^		
Final year of high school	109 (39.2)	212 (38.3)
Studying another course	62 (22.3)	106 (19.2)
Working	71 (25.5)	177 (32.0)
Undertaking a ‘gap year’	24 (8.6)	35 (6.3)
Other	6 (2.2)	9 (1.6)

*Note*: Data are *n* (%), unless otherwise specified.

^a^ Maximum missing data were for ‘Primary role in year prior to commencing program of study’ (podiatry, *n* = 6; non‐podiatry, *n* = 14). Missing data were for ‘Age’ (podiatry, *n* = 1), ‘Prior educational qualifications’ (podiatry, *n* = 1), ‘University’ (non‐podiatry, *n* = 1), ‘Year of study’ (non‐podiatry, *n* = 1), ‘International student’ (non‐podiatry, *n* = 1).

^b^ Part‐time study load was defined as completing one to two subjects or 30 credit points or less.

Nine first‐year podiatry students volunteered to participate in the workshops. The first workshop was conducted online on March 30, 2022 with four students from Australia (two females and two males) and the second workshop was conducted online on April 8, 2022 with five female students from New Zealand.

### 3.2 Motivators, influencers and perceptions to studying podiatry

Descriptive statistics from the podiatry student surveys found on a 4‐point Likert scale (1 = not at all, 4 = to a great extent) that the top‐rated (i.e., moderate to great extent) motivators for studying podiatry were ‘interest in a health‐related career’ (mean 3.7 ± 0.6), ‘wanting to make a difference to people's health’ (mean 3.6 ± 0.7), and the ‘opportunity to care for people from different backgrounds’ (mean 3.1 ± 1.0) (Table 2). The top‐rated influencer for podiatry students was ‘themselves’ (mean 3.7 ± 0.7) (Table 2). According to podiatry students, the top five factors that make podiatry an attractive profession include: (i) job prospects after graduation (mean 3.6 ± 0.6), (ii) ability to work in private practice (mean 3.5 ± 0.7), (iii) wide scope of practice (mean 3.5 ± 0.7), (iv) prospect of owning your own business (mean 3.4 ± 0.8) and (v) ability to be involved in different areas of the profession (mean 3.3 ± 0.8) (Table 2).

**TABLE 2:** Motivators and influencers for career choices among podiatry and non‐podiatry students.

Survey questions and responses	Podiatry (*n* = 264)		Non‐podiatry (*n* = 509)	
	Mean (SD)	Median (IQR)	Mean (SD)	Median IQR
To what extent did the following factors spark your interest in studying podiatry [health/sport/science course] (i.e., to what extent did these factors motivate you)?				
Interest in a health‐related career	3.69 (0.59)	4 (3, 4)	3.72 (0.52)	4 (4, 4)
Interest in a sport‐related career	‐	‐	2.08 (1.29)	2 (1, 3)
Wanted to make a difference to people's health	3.55 (0.72)	4 (3, 4)	3.73 (0.51)	4 (4, 4)^a^
Opportunity to care for people from different backgrounds and age groups	3.12 (0.97)	3 (2, 4)	3.24 (0.88)	3 (3, 4)
Inspired by a podiatrist [inspired by a health professional]	2.34 (1.34)	2 (1, 4)	2.80 (1.15)	3 (2, 4)^a^
Encouraged by a peer	1.83 (1.20)	2 (1, 3)	2.09 (1.12)	2 (1, 3)^a^
Encouraged by a family member	2.46 (1.24)	3 (1, 4)	2.49 (1.20)	3 (2, 4)
Earning potential	2.75 (0.97)	3 (2, 3)	2.53 (1.02)	3 (2, 3)^a^
Could not get into another course	1.38 (1.21)	1 (1, 2)	0.87 (0.80)	1 (0, 1)^a^
Availability of scholarships and financial assistance	0.90 (0.89)	1 (0, 1)	0.98 (0.85)	1 (0, 1)
Multiple career options post graduation	2.66 (1.24)	3 (2, 4)	2.78 (1.18)	3 (2, 4)
Flexible working hours	2.64 (1.17)	3 (2, 4)	2.31 (1.13)	2 (1, 3)^a^
When considering your future career path, to what extent did the following people influence your choice to study podiatry [health/sport/science course]?				
Myself	3.66 (0.67)	4 (3, 4)	3.86 (0.37)	4 (4, 4)^a^
Parent	2.45 (1.16)	3 (1.75, 3)	2.23 (1.11)	2 (1, 3)^a^
Family member	2.14 (1.18)	2 (1, 3)	2.06 (1.06)	2 (1, 3)
Spouse/Partner	1.12 (1.30)	1 (0, 2)	1.27 (1.32)	1 (0, 2)
Friend	1.57 (1.16)	1 (1, 2)	1.78 (1.02)	2 (1, 2)^a^
Career counsellor	0.94 (0.97)	1 (0, 1)	1.17 (0.93)	1 (1, 2)^a^
School teacher	0.91 (0.88)	1 (0, 1)	1.23 (0.92)	1 (1, 2)^a^
Podiatrist [health professional]	1.83 (1.39)	1 (1, 3)	2.03 (1.22)	2 (1, 3)^a^
Health professional (other than a podiatrist)	1.66 (1.27)	1.5 (1, 3)	‐	‐
Podiatry student [health/sport/science student]	1.28 (1.15)	1 (0, 2)	1.27 (1.01)	1 (1, 2)
Recent podiatry graduate (within 3 years) [recent health/sport/science graduate]	1.14 (1.10)	1 (0, 1)	1.17 (1.04)	1 (1, 1)
Sporting coach	‐	‐	0.95 (0.86)	1 (0, 1)
In your opinion, to what extent do you think the following factors make podiatry [health/sport/science course] an attractive profession?				
Job prospects after graduation	3.56 (0.61)	4 (3, 4)	3.48 (0.67)	4 (3, 4)
Wide scope of practice	3.40 (0.68)	3 (3, 4)	3.58 (0.64)	4 (3, 4)^a^
Ability to be involved in different areas of the profession (e.g. clinical, teaching, research)	3.27 (0.84)	3 (3, 4)	3.35 (0.79)	4 (3, 4)
Prospect of owning your own business	3.37 (0.85)	4 (3, 4)	2.66 (1.11)	3 (2, 4)^a^
Offers pathways into other health or science disciplines	2.53 (1.09)	2 (2, 4)	2.21 (1.00)	2 (1, 3)^a^
Sports and rehabilitation podiatry speciality	3.05 (0.97)	3 (2, 4)	‐	‐
High‐risk foot podiatry speciality	3.05 (0.96)	3 (2, 4)	‐	‐
Paediatric podiatry speciality	2.93 (0.99)	3 (2, 4)	‐	‐
Podiatric surgery speciality (i.e., minor skin and nail procedures)	3.12 (0.94)	3 (3, 4)	‐	‐
Pathways for endorsement of scheduled medicines	2.85 (1.03)	3 (2, 4)	‐	‐
Pathways to become a podiatric surgeon	2.98 (1.08)	3 (2, 4)	‐	‐
Ability to work in hospitals	3.14 (0.94)	3 (3, 4)	2.88 (1.00)	3 (2, 4)^a^
Ability to work in community health	3.07 (0.95)	3 (2, 4)	3.05 (0.84)	3 (3, 4)
Ability to work in private practice	3.53 (0.66)	4 (3, 4)	3.18 (0.86)	3 (3, 4)^a^
International employment opportunities	3.04 (1.01)	3 (2, 4)	2.90 (1.05)	3 (2, 4)

*Note*: Questions were answered on a 4‐point Likert scale (1, not at all; 2, to a small extent; 3, to a moderate extent; 4, to a great extent). Text outlined in square brackets corresponds to questions or responses specific to the non‐podiatry student survey.

Abbreviations: IQR, interquartile range; SD, standard deviation.

^a^ Indicates a significant between‐group difference (*p* ≤ 0.05) based on Mann‐Whitney *U* test.

When comparing the podiatry and non‐podiatry student responses, the motivators and influencers which inform students' respective career choices and what they perceive as attractive about their profession, differed between the two groups. Table 3 presents the results of the multiple logistic regression analyses. Compared to the non‐podiatry students, podiatry students were *more likely to be motivated* to study podiatry if they failed to get into another course (odds ratio [OR] 1.59, 95% confidence interval [CI] 1.34 to 1.90) or by the flexible working hours (OR 1.54, CI 1.29 to 1.85). Podiatry students were *less likely to be motivated* by a health professional (OR 0.79, CI 0.68 to 0.91) or peer (OR 0.81, CI 0.68 to 0.96), the multiple career options post graduation (OR 0.82, CI 0.70 to 0.96), wanting to make a difference to people's health (OR 0.67, CI 0.48 to 0.94) or the availability of scholarships (OR 0.74, CI 0.60 to 0.91) (Table 3).

**TABLE 3:** Multiple logistic regression analysis exploring *motivators, influencers* and *perceptions* associated with podiatry career choice.

Predictors	Motivators			Influencers			Perceptions		
	OR	95% CI	*p*‐value^a^	OR	95% CI	*p*‐value^a^	OR	95% CI	*p*‐value^a^
(Intercept)	0.74	0.18 to 3.05	0.677	11.72	2.64 to 55.61	0.002	0.19	0.05 to 0.74	0.017
Age	0.99	0.97 to 1.02	0.620	0.99	0.97 to 1.02	0.597	1.00	0.97 to 1.02	0.727
Female sex	0.56	0.39 to 0.81	0.002^a^	0.61	0.43 to 0.87	0.006^a^	0.71	0.48 to 1.03	0.072
Prior educational qualifications	1.30	0.88 to 1.92	0.188	1.71	0.16 to 2.52	0.007^a^	1.33	0.89 to 2.00	0.165
Interest in health‐related career	1.24	0.88 to 1.76	0.220						
Make a difference to people's health	0.67	0.48 to 0.94	0.020^a^						
Opportunity to care for people from different backgrounds and age groups	1.11	0.90 to 1.37	0.336						
Inspired by a health professional	0.79	0.68 to 0.91	0.002^a^						
Encouraged by a peer	0.81	0.68 to 0.96	0.016^a^						
Encouraged by a family member	1.10	0.94 to 1.29	0.244						
Earning potential	1.18	0.98 to 1.43	0.078						
Could not get into another course	1.59	1.34 to 1.90	<0.001^a^						
Availability of scholarships and financial assistance	0.74	0.60 to 0.91	0.005^a^						
Multiple career options	0.82	0.70 to 0.96	0.013^a^						
Flexible working hours	1.54	1.29 to 1.85	<0.001^a^						
Myself				0.51	0.36 to 0.71	<0.001^a^			
Parent				1.34	1.10 to 1.62	0.003^a^			
Family member				1.01	0.83 to 1.23	0.900			
Spouse/Partner				0.96	0.84 to 1.11	0.598			
Friend				0.86	0.72 to 1.01	0.065			
Career counsellor				0.91	0.73 to 1.14	0.416			
School teacher				0.66	0.51 to 0.84	0.001^a^			
Health professional				0.77	0.67 to 0.89	<0.001^a^			
Health student				1.24	1.03 to 1.49	0.026^a^			
Health graduate (within 3 years)				1.09	0.91 to 1.30	0.363			
Job prospects							1.43	1.01 to 2.05	0.050^a^
Wide scope of practice							0.38	0.27 to 0.54	<0.001^a^
Ability to work in different areas of profession							0.80	0.62 to 1.04	0.100
Owning own business							2.15	1.73 to 2.69	<0.001^a^
Career pathways (into other health disciplines)							1.08	0.90 to 1.29	0.398
Work in hospitals							1.48	1.18 to 1.87	0.001^a^
Work in community health							0.84	0.65 to 1.07	0.156
Work in private practice							1.21	0.90 to 1.63	0.208
International employment							1.10	0.91 to 1.33	0.318

*Note*: Motivators model based on 772 observations; Influencers model based on 759 observations; Perceptions model based on 735 observations.

Abbreviations: CI, Confidence interval; OR, Odds ratio.

^a^ Significant difference between podiatry and non‐podiatry students (*p* ≤ 0.05).

Podiatry students were *more likely to be influenced* to study podiatry if they had prior education/qualifications (OR 1.71, CI 0.16 to 2.52) and *less likely* if they were female (OR 0.61, CI 0.43 to 0.87). Parents (OR 1.34, CI 1.10 to 1.62) and current health students (OR 1.24, CI 1.03 to 1.49) were *more likely to influence* podiatry students, while school teachers (OR 0.66, CI 0.51 to 0.84), health professionals (OR 0.77, CI 0.67 to 0.89) or students themselves (OR 0.51, CI 0.36 to 0.71) were *less likely to influence* podiatry students as compared to non‐podiatry students (Table 3).

When comparing *perceptions* of factors which make a profession attractive, podiatry students were *more likely* to think that job prospects after graduation (OR 1.43, CI 1.01 to 2.05), the prospect of owning own business (OR 2.15, CI 1.73 to 2.69) and the ability to work in hospitals (OR 1.48, CI 1.18 to 1.87) were attractive factors, while the wide scope of practice (OR 0.38, CI 0.27 to 0.54) was *less* attractive, compared to the non‐podiatry students (Table 3).

### 3.3 Barriers to studying podiatry

Students commonly encountered barriers when choosing to study podiatry. Overall, 28.1% of podiatry students encountered barriers, while 19/50 (38.0%) of non‐podiatry students encountered barriers when they initially considered studying podiatry. Podiatry students were more likely to encounter barriers if they had prior education/qualifications (OR 2.12, CI 1.03 to 4.35), while non‐podiatry students were more likely to encounter barriers if they had carer responsibilities (OR 17.94, CI 1.71 to 556.33) (Table 4). Examples of barriers for the podiatry students included negative stereotypes and perceptions of the podiatry profession, work–life balance and poor awareness of the podiatry profession. Barriers are further explored within the qualitative data analysis.

**TABLE 4:** Barriers to studying podiatry among podiatry and non‐podiatry students.

Predictors	Podiatry			Non‐podiatry		
	OR	95% CI	*p*‐value^a^	OR	95% CI	*p*‐value^a^
(Intercept)	2.47	0.35 to 18.27	0.367	1.23	0.02 to 182.09	0.926
Age	0.98	0.93 to 1.02	0.315	0.96	0.81 to 1.08	0.541
Female sex	0.91	0.48 to 1.74	0.782	2.30	0.57 to 10.93	0.256
Carer responsibilities	1.84	0.70 to 4.86	0.215	17.94	1.71 to 566.33	0.043^a^
Prior educational qualifications	2.12	1.03 to 4.35	0.041^a^	0.48	0.05 to 3.04	0.459
Full‐time student	0.72	0.24 to 2.26	0.562	0.64	0.06 to 7.69	0.712
International student	2.85	0.94 to 8.96	0.066			

*Note*: Podiatry student model based on 253 observations (*R* ^2^ Tjur = 0.121); Non‐podiatry student model based on 50 observations (*R* ^2^ Tjur = 0.167).

Abbreviations: CI, Confidence interval; OR, Odds ratio.

^a^ Significant difference (*p* ≤ 0.05).

**FIGURE 1:**
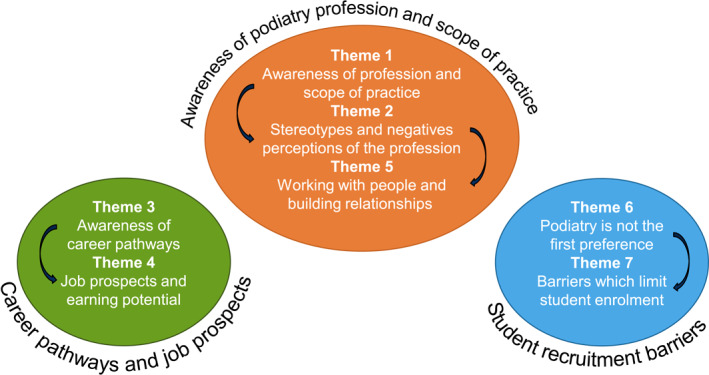
Thematic map of motivators and barriers for studying podiatry.

The themes presented in this paper represent contributions from podiatry and non‐podiatry students, with a primary focus on the motivators and barriers for studying podiatry. However, the themes also illuminate differences between the perceptions of podiatry and non‐podiatry students in relation to their respective career choices. The inter‐relationships between these seven themes are illustrated in Figure 1. Three over‐arching issues are identified as fundamental to understanding the motivators and barriers for studying podiatry: (i) awareness of the podiatry profession and scope of practice; (ii) career pathways and job prospects; and (iii) student recruitment barriers.

### 3.4 Theme 1. Awareness of profession and scope of practice

This theme provides insight into the students' lack of understanding of the podiatry profession and its scope of practice, compared to other health disciplines. Students perceived a relatively narrow scope of practice in podiatry when compared to other healthcare disciplines:

*…it feels like a small field with not a lot of scope for change and/or movement*

(participant #695, female (F), fourth‐year occupational therapy student).

For two participants, there was a desire to have a more holistic approach, involving the entire body, rather than joining a profession that is perceived to primarily focus on the foot. This reflection limited the appeal of the profession:

*It [podiatry] doesn't align with my desire to work in a field where the person is viewed holistically as opposed to a particular body part*

(participant #695, F, fourth‐year occupational therapy student).

An occupational therapy student described what attracted them to their discipline including the reputation that occupational therapists are involved in more innovative and holistic problem‐solving solutions. This is not a typical perception of podiatry:

*It [podiatry] is less holistic and more clinical than occupational therapy. This broad problem solving/creativity aspect of occupational therapy is what I was ultimately drawn to*

(participant #99, F, third‐year occupational therapy student).

In contrast to the views of non‐podiatry students, quantitative data found that for podiatry students a broad scope of practice was a factor that makes podiatry an attractive profession. In response to the question ‘*To what extent do you think the following factors make podiatry an attractive profession?*’ scope of practice was rated ‘to a moderate extent’ (mean 3.4 ± 0.7), on a 4‐point Likert scale (1 = not at all, 4 = to a great extent). These data were complemented by the voice of podiatry students. One student described the role of podiatrists across broad medical domains in healthcare but noted that this is not recognised by the public:

*I believe the work we do is not communicated properly. The work we do can overlap with the integumentary, muscular, cardiovascular and neurological fields of medicine, yet there is no recognition of this in the general public*

(workshop #1, first‐year podiatry student).

Podiatry students emphasised that podiatric practice is extensive and goes beyond simply addressing foot‐related issues. These students appreciated the opportunity to combine their interests in sports and biomechanics with the interpersonal and compassionate aspects of providing healthcare:

*Podiatry incorporated the sport and biomechanical side of studies with the people side of allied health, which suits my personality type and interests*

(workshop #1, first‐year podiatry student).

The quotes above reveal a lack of awareness about the role of a podiatrist, particularly amongst non‐podiatry students. However, one international student noted a lack of awareness about podiatry, particularly among people from countries where podiatry is not a recognised profession. This highlights an important consideration that podiatry, as an allied health profession, might be unfamiliar to people, including those who might benefit from podiatry services:

*Podiatry is only recognised in a few countries such as Australia, New Zealand, the UK, the US and Canada. It does not exist in other countries so people from overseas apart from those countries may not know what podiatry is. I also did not know what it was at first and people I know, or people from similar background to mine (I have an Asian background) did not know about it either*

(participant #339, male (M), third‐year podiatry student).

Some participants emphasised the profession's need to raise awareness about its diverse scope of practice. They believed that by doing so, more individuals might become interested in studying podiatry. This is explored further within themes two to seven.

### 3.5 Theme 2. Stereotypes and negative perceptions of the profession

This theme illustrates student experiences and their perceptions of the podiatry profession. Several participants described unappealing stereotypes of the profession including the perception that podiatrists primarily perform toenail and skin procedures for older clients and/or manage skin disease and wounds:

*Why would anyone want to work in podiatry? Imagine introducing yourself to strangers: ‘hello, yes, I play with diabetic feet all day and oh yes, yesterday I dug out a corn! Life is splendid!’. No. Just no*

(participant #143, M, first‐year nursing student).

*Experiences of colleagues who leave the field within five years due to repetitive unrewarding age‐related work ‐ trimming and grinding nails and calluses*

(participant #494, M, second‐year physiotherapy student).

The negative stereotypes were associated with a perception that podiatry may be less valued than other healthcare professions *“…[podiatry profession] has not been given the respect it deserves”* and *“…it [podiatry profession] has a bad reputation within the population as not being a real health profession”* (participant #210, F, second‐year podiatry student).

One podiatry student expressed frustration and disappointment about the misconceptions of podiatry but also a strong sense of advocacy, which involved emphasising the depth and expertise of the profession:

*People including a GP asking me why I'd pick such a field as all we do is "cut nails" ‐ their comments upset me greatly as the field of podiatry is far more than just that. We are specialists of the lower limbs!*

(participant #634, F, fourth‐year podiatry student).

One student also expressed disillusionment about podiatry's reputation and the quality of podiatrists entering the profession, and specifically referred to the Australian Tertiary Admission Rank (ATAR) requirements for an undergraduate podiatry degree with Australia:

*I've heard too many negative remarks about podiatrists. I think the ATAR [Australian Tertiary Admissions Rank] requirements are way too low and have allowed people to graduate to a really low standard. I believe this has allowed low quality to enter the workforce thus subsequently affecting the overall stereotype of podiatrists*

(participant #788, M, third‐year physiotherapy student).

Some podiatry students displayed a strong determination and motivation to change the negative stereotypes linked to their profession and a commitment to alter the public perception of podiatry by emphasising the valuable role that podiatrists play in healthcare:

*The public do not understand the importance of foot health and the positive impact this profession has on people's lives. If we can change the stigma around podiatry, and what we actually do ‐ this may attract more students*

(participant #476, F, fourth‐year podiatry student).

### 3.6 Theme 3. Awareness of career pathways

This theme highlights the students' poor understanding of podiatry career pathways. While the scope of podiatry practice is not clear to people outside of the podiatry profession, there is a similar lack of understanding about career opportunities. This general lack of awareness of podiatry as a possible career pathway creates a barrier for potential students who have an interest in healthcare. Many students stated that they were not interested in podiatry or did not have enough knowledge about the profession to consider it as a career option:

*To be honest, despite having been to a podiatrist numerous times in my life and needing to wear orthotics… I don't have a clue about career options, or what or how long the training would take*

(participant #515, F, second‐year occupational therapy student).

It is apparent that prospective students are unlikely to choose podiatry as a career option if they do not understand the occupation, or where they might practice:

*… understanding all the elements that make up the study of podiatry would have been more of a driving factor for the decision, in that I could explore fields such as dermatology, sports rehab, community care*

(workshop #1, first‐year podiatry student).

A general lack of insight into the profession, compounded by a lack of information relating specifically to podiatry practice, creates a risk that prospective students will make career decisions without understanding all the opportunities available to them:

*I am unsure about what clients or environments a podiatrist could work with/in which are the questions that I asked before choosing my current program (OT)*

(participant #511, F, second‐year occupational therapy student).

Compared to students enrolled in other health disciplines, podiatry students were more likely to be motivated by ‘flexible working hours’ (OR 1.54, CI 1.29 to 1.85). In addition, the ‘ability to work in hospitals’ (OR 1.48, CI 1.18 to 1.87) was an attractive aspect of the profession for podiatry students. With respect to career pathways, podiatry student respondents considered the ability to work in private practice (mean 3.53 ± 0.66), hospitals, (mean 3.14 ± 0.94) or within different areas (e.g., clinical, teaching, research), (mean 3.27 ± 0.84) were attractive factors of the podiatry profession.

### 3.7 Theme 4. Job prospects and earning potential

This theme highlights that prospective students may not always have accurate knowledge to inform decision‐making about their higher education choices and career planning. A low awareness of job prospects, employment opportunities and earning potential were identified as inter‐related factors that deter students from considering podiatry as a potential career pathway. Students highlighted the importance of raising these issues to prospective students:

*Talk about career prospects and employment rates. That was what solidified my decision to pursue podiatry. Also, mention how broad the podiatry field is (e.g., paediatrics, high risk podiatry, aged care, education etc). I feel like that’s not common knowledge and it would be important to know how broad the field is for people considering podiatry*

(participant #129, F, first‐year podiatry student).

Misinformation may be conveyed to prospective students from a range of sources. A non‐podiatry student *“assumed [podiatry] to have limited job opportunities”* (participant #107, F, orthoptics student). Similarly, current podiatry students shared their experiences:

*Job availability [in podiatry]….people would tell me it was a dying field*

(participant #634, F, fourth‐year podiatry student)

*During placement I found out from a few podiatrists the pay was barely above minimum wage*

(participant #684, F, third‐year podiatry student).

Another student expressed a desire for a career offering multiple job opportunities and the ability to work in various settings after graduation. A career in podiatry was perceived to be limited to working in a clinical setting, whereas other professions, such as occupational therapy and physiotherapy, were seen as offering more diverse work environments:

*Too clinical and not enough opportunities to work [out] of a clinical practice…*

(participant #348, F, third‐year physiotherapy student)

Interestingly, podiatry respondents rated the ‘ability to be involved in different areas of the profession (e.g., clinical, teaching, research)’ to ‘a moderate extent’ (mean 3.27 ± 0.84) when asked what makes podiatry an attractive profession. In addition, ‘job prospects after graduation’ (mean 3.56 ± 0.61) and ‘the prospect of owning your own business’ (mean 3.37 ± 0.85) were two highly rated factors that podiatry students considered ‘to a great extent’, which makes podiatry an attractive profession. This finding was further supported by logistic regression analyses. Compared to students in other health disciplines, podiatry students were more likely to view their profession as attractive due to the ‘job prospects after graduation’ (OR 1.43, CI 1.01 to 2.05) and the ‘prospect of owning your own business’ (OR 2.15, CI 1.73 to 2.69). Furthermore, podiatry students considered international employment opportunities to be an attractive aspect of the profession (mean 3.0 ± 1.0).

Given these positive perceptions, it is important that all stakeholders who influence prospective students' decision‐making and career planning provide insight into the potential breadth of opportunities in podiatry. As one student reflected:

*[I] didn't originally know much about the job prospects, as well as not knowing in depth what the role of a podiatrist entails, including how broad the profession stretches across multiple disciplines (i.e., sports, paediatrics, podiatric surgery etc*.*)*

(participant #104, F, second‐year podiatry student).

### 3.8 Theme 5. Working with people and building relationships

This theme provides insight to the altruistic reasons influencing a student's choice to study podiatry. A strong theme emerged about the motivation of students who considered studying podiatry, or were enrolled in a podiatry program, about working with people. Many participants expressed the joy of *“interacting with new patients all day every day and building relationships”* (workshop #1, first‐year podiatry student). Some participants described the *“varied practice settings and client groups – age/gender/culture/health conditions”* (workshop #1, first‐year podiatry student) that a podiatry student encounters in their training, which reflects the diversity of a podiatry practice and vast opportunities for interpersonal interactions with patients.

Several students discussed the prospect of enhancing patient well‐being, and that podiatry aligned with their personal interests, which influenced their decision to study podiatry. Students emphasised that as podiatry is a smaller discipline, they felt supported and were able to build on relationships with educators and fellow students during their course and it encouraged hands‐on learning:

*It's more hands on, because of the class size we are able to get more support and feel more comfortable asking for help and building relationships as well*

(workshop #2, first‐year podiatry student).

Quantitative data supported the qualitative findings. In response to the question *‘To what extent did the following factors spark your interest in studying podiatry?’*, the ‘opportunity to care for people from different backgrounds and age groups’ was responded on average ‘to a moderate extent’ (mean 3.12 ± 0.97), and ‘wanted to make a difference to people's health’ was rated 3.55 ± 0.71 on a 4‐point Likert scale.

### 3.9 Theme 6. Podiatry is not the first preference

This theme highlights the need for further attention to podiatry program marketing and student recruitment activities. It is common for high school students to report that enrolling in podiatry was not their first choice. In this study, the podiatry students were more likely to be motivated to choose podiatry if they failed to get into another course (OR 1.59, CI 1.34 to 1.90), in comparison to students in other health disciplines. From a total of 253 podiatry students, 98 (38.7%) indicated that podiatry was not their first preference when applying to study. However, students may be interested in the opportunity that podiatry provides for specialisation in healthcare:

*I did not get into medicine, and I believed that this course would give me a very similar, if not the same experience, as being a medical student*

(workshop #1, first‐year podiatry student).

*[I] wanted to do something within health care that involved one specific part of the body. I originally wanted to do optometry however I did not get in*

(workshop #2, first‐year podiatry student).

If a prospective student has the opportunity for work experience in a podiatry clinic where they can observe a podiatrist/podiatry student, and gain insight to the scope of practice, this is a powerful influence on their study planning. Similarly, prior experience of podiatry as a client may be the trigger for a student to choose this career path:

*I saw a podiatrist six years ago who helped me with a football injury. I knew I wanted to do something in healthcare but wanted a degree which focused on lower limb injuries*

(workshop #2, first‐year podiatry student).

*…opportunities for prospective students, and students considering transferring courses, to attend a class or placement so they can decide if podiatry is the right career path for them*

(participant #248, M, sport and exercise science).

In comparison to students in other health disciplines, podiatry students were more likely to be influenced to study podiatry if they had completed prior education or gained another qualification (OR 1.71, CI 0.16 to 2.52). This is relevant to consider in relation to podiatry program marketing and student recruitment activities.

### 3.10 Theme 7. Barriers which limit student enrolment

This theme discusses some of the key barriers to studying podiatry, specifically related to access, international students and prior education challenges. At the time of data collection for this study, podiatry programs were being offered at 10 universities located in five states within Australia and one program was being delivered in Auckland, New Zealand. Most of these institutions are in metropolitan centres and the podiatry programs require students to enrol for on‐campus study. This program delivery model does limit access for prospective students, particularly those living in regional, rural and remote locations:

*When I first finished year 12, podiatry was my number one preference and while I was accepted, the location of the uni[versity] made it difficult for me to go and study*

(participant #50, F, fourth‐year physiotherapy student).

Lack of online components, distance to uni[versity]

(participant #161, F, second‐year podiatry student).

In many countries, podiatry is not offered as a university program and prospective students may need to travel internationally to study podiatry. International students may face financial barriers that may not affect domestic students:

*Podiatry is not offered in my country which meant that I was forced to study in Australia, thus the financial burden is too high*

(participant #376, F, second‐year podiatry student).

Mature‐age students may face barriers to enrolling in a podiatry program compared with students from high school, such as lack of confidence in their academic capability and/or carer responsibilities. Many institutions have bridging courses that provide an access pathway for mature‐age students who do not meet the program entry requirements. However, this may add several months of study for the prospective student and presents another barrier to studying podiatry:

*Older age, already full‐time working, wanting to buy property, unsure if to complete another 4‐year undergraduate or 2‐year masters…*

(participant #114, F, fourth‐year podiatry student).

Discipline‐specific content within the podiatry curriculum also means that individuals from health‐related professions (e.g., nursing), may not receive recognition of prior knowledge based on their occupation and be required to enrol from first year. This may influence prospective students' decision to change career to podiatry:

*The uni[versity] did not recognise my previous occupation as I am a mature age student and it was quite difficult getting accepted*

(participant #310, F, third‐year podiatry student).

Quantitative data supported the qualitative findings. In response to the question ‘*When considering podiatry as a career, did you encounter any barriers (or was there anything that may have deterred you) from choosing to study podiatry?*’, 28.1% of podiatry students encountered barriers when applying to study. Interestingly, podiatry students were more likely to encounter barriers if they had prior education/qualifications (OR 2.12, CI 1.03 to 4.35). Of the 50 non‐podiatry students that considered studying podiatry, 19 (38.0%) encountered barriers, which was more likely if they had carer responsibilities (OR 17.94, CI 1.71 to 566.33).

Since many podiatry students are mature‐age students, with approximately 26% having work and 13% with carer responsibilities, they may face more challenges during their course. When podiatry students were asked ‘*Throughout your studies in podiatry, have you ever thought about leaving the course?*’, over a third of students (37.5%; *n* = 95/253) had considered leaving their podiatry course. This was higher than the non‐podiatry students (31.1%; *n* = 147/473). The most common reasons why podiatry students considered leaving their course were related to ‘health or stress’ (50.5%; *n* = 48/95), ‘study/life balance’ (45.3%; *n* = 43/95) and ‘difficulties relating to workload’ (36.8%; *n* = 35/95).

## 4 DISCUSSION

This is the first mixed methods study to explore the motivators and barriers for studying podiatry in Australia and New Zealand, with direct comparisons made to relevant health, sport and science students and their respective career choices. This discussion illuminates potential opportunities for a broad range of stakeholders to address the three over‐arching issues illustrated in Figure 1, to ensure future sustainability of podiatry education programs and the podiatry workforce.

### 4.1 Awareness of podiatry profession and scope of practice

There are a variety of internal and external factors that both motivate and influence prospective podiatry students. This study found that an *interest in a health‐related career*, *wanting to make a difference to people's health* and the *opportunity to care for people from different backgrounds and age groups* were highly motivating factors among podiatry students. Our findings are supported by previous literature [20, 21, 22], whereby altruistic reasons are the main motivators for studying podiatry.

Similar to the UK [20, 21], there is an apparent lack of understanding about the podiatry profession amongst students, particularly relating to scope of practice, career pathways/opportunities, job prospects and earning potential. It is difficult to ascertain specific reasons for this lack of understanding, but it is reasonable to suggest a failure across the podiatry profession to educate the public and other health professionals about its development over decades and the current scope of practice.

Australian podiatrists typically collaborate with a median of three allied health professionals in their main workplace [6], limiting broad interdisciplinary opportunities. This challenge is likely to be more pronounced for the majority of podiatrists in Australia and New Zealand who work in private practices [6, 8], where opportunities for multidisciplinary collaboration are likely to be fewer compared to those working in public health settings, particularly large metropolitan hospitals. Furthermore, a recent survey of Australian podiatrists (*n* = 1135) showed that only 175 podiatrists (15%) planned to progress through the Australian Podiatry Association Career Framework [6]. As a result, most podiatrists are not extending their scopes of practice through the Career Framework and endorsement for scheduled medicines, thereby not fully demonstrating the breadth of work practice possibilities within the profession. When considering the awareness of the podiatry profession in the general community, it is crucial to address the continuing prevalence of negative stereotypes. Consistent with previous studies [20, 21], negative perceptions of the podiatry profession were evident among non‐podiatry students. However, podiatry students were more likely to highlight the positive aspects of the discipline.

While this study did not seek to understand the beliefs held by students which inform their perceptions of podiatry, it is interesting to consider why a student might believe that a podiatrist would not view a patient holistically (i.e., podiatrists focus only on foot‐related pathology rather than the overall health of the patient). This perception of podiatry was a significant source of frustration to current podiatry students, many of whom demonstrated strong advocacy for challenging these negative stereotypes.

However, considering that many non‐podiatry healthcare roles also involve tasks which might be viewed negatively by prospective students, it does suggest that the podiatry profession has been less successful in promoting the positive aspects of podiatry practice and the opportunities for a podiatrist to make a critical impact on a person's health and wellbeing.

### 4.2 Career pathways and job prospects

While podiatry may not be the first preference for a prospective student, podiatry students did suggest a variety of factors that make it an attractive career option. Factors including *job prospects after graduation*, *the ability to work in private practice*, *broad scope of practice*, *the prospect of owning your own business* and the *ability to be involved in different areas of the profession* were the most highly rated factors among the podiatry students. Due to the divergent findings within the qualitative data obtained from the non‐podiatry students, it seems apparent that non‐podiatry students have a limited understanding of podiatry career pathways and job prospects. It remains unknown if podiatry students shared the same views prior to enrolling in their course, of if they had a better understanding and this contributed to them choosing to study podiatry.

Although this study demonstrated similarities between the podiatry and non‐podiatry students for the motivators and barriers for their career choices, there are distinct differences between these two cohorts particularly in their perceptions about what makes their profession attractive. Evidence of poor knowledge among the non‐podiatry students relating to job prospects, employment opportunities and earning potential for podiatrists is of high concern, considering the current workforce needs and shortfall of podiatry service provision, particularly in rural and remote locations [18].

### 4.3 Student recruitment barriers

In addition to the general lack of understanding about the podiatry profession, career opportunities and job prospects, additional barriers for some students means that podiatry is not their first preference for university study. For some students, travel or relocation to study on campus is not feasible. Currently, podiatry programs are offered at only one university in New Zealand and nine universities across eight cities in Australia. As a result, students living outside these locations face the options of relocating, travelling or forgoing the opportunity to study podiatry altogether. Additionally, financial constraints are increasingly relevant, particularly for international students, and many prospective students have employment commitments and/or carer responsibilities which compete with a full‐time study workload.

The structure of podiatry programs is of current interest to universities with the opportunity to combine on‐campus and online study for hybrid program delivery. Designing flexibility into a podiatry program does address the geographical barriers and potentially some of the financial constraints for prospective students. Target groups for the promotion of podiatry programs should also extend beyond high school students to include people considering a change of career. Podiatry should also be offered as an alternative career choice for students who are unsuccessful in their first preference (e.g., medicine, dentistry or physiotherapy). Considering the finding that students with prior degrees might be interested in studying podiatry but encounter barriers to enrolment (such as those related to mid‐career changes), future research and marketing strategies should explore methods to enhance recruitment potential among this group of people.

Financial barriers, as identified in this study, seemed to disproportionately affect international students. The high cost of study for an international student limits access to those with a higher socio‐economic status. The Australian Dollar and the New Zealand Dollar are currently favourable compared to currencies such as the UK pound, which makes Australia and New Zealand preferred study destinations for students from some Asian countries. However, with inflation and rising costs of living, this situation may change within the next few years.

## 5 LIMITATIONS AND STRENGTHS

This study needs to be viewed considering some limitations. First, some of the closed‐ended questions in the online survey may have limited deeper responses from participants. However, free‐text options were included wherever possible. Additionally, the incorporation of qualitative workshops allowed for further exploration of student experiences and perceptions. Second, no survey responses were received from the dietetics/nutrition discipline. However, this is unlikely to have limited the scope of responses as the student participants represented a wide range of health, sport and science disciplines (13 in total). Third, there were missing data for some of the survey responses due to students only completing the demographic section of the survey before exiting. To account for this, all linear and logistic regression models included participants with completed data. Fourth, while the survey component of our study was initially piloted on first‐year podiatry students at three universities (*n* = 30), the survey was not validated. Fifth, only two of the scheduled workshops were conducted, which may have limited comparisons between student cohorts at the participating universities. Recruitment was impacted by the COVID‐19 pandemic and competing educational requirements at the time. To ensure an adequate group size for discussion, only two of the four workshops were implemented. Sixth, we did not record ethnicity, including Indigenous status, of the study participants. Therefore, we cannot be confident that our findings reflect the experience of all cultural groups within Australia and New Zealand. The decision to exclude Indigenous status was made to protect the anonymity of respondents. Due to the low percentage of Indigenous students enrolled in Australian podiatry courses, including this information alongside data on university affiliation and year level could have compromised respondent confidentiality. Therefore, to maintain the privacy of all participants, we omitted Indigenous status from our study. This omission limits the generalisability of the findings.

This is the first mixed methods study to explore motivators and barriers for studying podiatry, with direct comparisons made to relevant non‐podiatry students and their respective career choices. A strength of our study is the large sample size (*n* = 278 for podiatry students and *n* = 553 for non‐podiatry students), which accounts for approximately 32% of podiatry students across Australia and New Zealand. In addition, integrating the quantitative and qualitative data allowed for the identification of key themes such as awareness of the podiatry profession, stereotypes and negative perceptions, career pathways, job prospects, working with people and barriers to student enrolment. These themes emerged from both the quantitative analysis and qualitative exploration, enriching the overall understanding of the topic. Furthermore, integration of both methods revealed complex relationships among variables. For instance, qualitative data provided insights into the association between stereotypes, negative perceptions and career choices, which might not have been fully captured through quantitative measures alone. Therefore, this data provides robust baseline data against which future studies and improvements from marketing initiatives can be benchmarked and monitored. Student comparisons may also be drawn between other countries.

### 5.1 Implications of study findings

The outcomes of this work will inform the promotion of podiatry programs and may contribute to increasing student enrolments, helping to sustain and expand the podiatry workforce. All sectors of the podiatry profession must work together to address negative stereotypes and develop potential strategies to amplify the positive aspects of podiatry as an essential allied health discipline. Promotion of the profession and current scope of practice could be achieved via professional networks, associations and universities. Further, podiatry practitioners should be encouraged to actively recruit the next generation of podiatrists through promotion and advocacy efforts. This study will also serve as a useful resource to inform the promotion of podiatry programs by Australian and New Zealand universities and will support key podiatry profession stakeholders (e.g., Australian Podiatry Association, Podiatry NZ) to design effective marketing campaigns. Consequently, this may increase university enrolments in podiatry and will likely have a broader impact on improving the stability and sustainability of the podiatry profession. This includes fostering continued research and innovation, podiatry advocacy at the government‐level, meeting patient demand, improving occupation share of employment, and ensuring the ongoing evolution of the profession's scope of practice.

## 6 CONCLUSIONS

This study identified three over‐arching issues as fundamental to understanding the motivators and barriers for studying podiatry: (i) awareness of podiatry profession and scope of practice; (ii) career pathways and job prospects; and (iii) student recruitment barriers. There is limited understanding of the podiatry profession among allied health students. Further work is required to reverse the negative stereotypes and perceptions of podiatry and build knowledge of the profession's scope of practice, career pathways/opportunities, job prospects and earning potential.

## AUTHOR CONTRIBUTIONS

**Michelle R. Kaminski**: Conceptualisation; formal analysis; funding acquisition; investigation; methodology; project administration; visualisation; writing—original draft; writing—review & editing. **Glen A. Whittaker**: Conceptualisation; formal analysis; investigation; methodology; writing—review & editing. **Caroline Robinson**: Conceptualisation; formal analysis; investigation; methodology; writing—original draft; writing—review & editing. **Matthew Cotchett**: Conceptualisation; formal analysis; investigation; methodology; writing—original draft; writing—review & editing. **Malia Ho**: Conceptualisation; formal analysis; investigation; methodology; writing—original draft; writing—review & editing. **Shannon E. Munteanu**: Conceptualisation; investigation; methodology; writing—review & editing. **Mollie Dollinger**: Investigation; writing—review & editing. **Sia Kazantzis**: Investigation; writing—review & editing. **Xia Li**: Formal analysis; writing—review & editing. **Ryan S. Causby**: Conceptualisation; investigation; writing—review & editing. **Mike Frecklington**: Conceptualisation; investigation; writing—review & editing. **Steven Walmsley**: Conceptualisation; investigation; writing—review & editing. **Vivienne Chuter**: Conceptualisation; investigation; writing—review & editing. **Sarah L. Casey**: Conceptualisation; investigation; writing—review & editing. **Burke Hugo**: Conceptualisation; investigation; writing—review & editing. **Daniel R. Bonanno**: Conceptualisation; formal analysis; investigation; methodology; project administration; writing—review & editing.

## CONFLICT OF INTEREST STATEMENT

The authors declare that they have no competing interests.

## ETHICS STATEMENT

This study was approved by the La Trobe University Human Research and Ethics Committee (HEC21057) and ethics approval was also obtained from the following institutions via mutual acceptance applications: Auckland University of Technology (21/161), Central Queensland University (22978), Charles Sturt University (H21077), Southern Cross University (2021/043), University of Newcastle (H‐2021‐0276), University of South Australia (203889), University of Western Australia (2021/ET000372) and Western Sydney University (H14404). All participants provided informed consent prior to data collection.

## Supporting information

Supporting Information S1

Supporting Information S2

Supporting Information S3

Supporting Information S4

## Data Availability

All data generated or analysed during this study are included in this published article.

## ENDNOTE

^1^
Auckland University of Technology (NZ); Central Queensland University (QLD); Charles Sturt University (NSW); La Trobe University (VIC); Queensland University of Technology (QLD); Southern Cross University (QLD); University of Newcastle (NSW); University of South Australia (SA); University of Western Australia (WA); and Western Sydney University (NSW).
