# Supplementary material for: Motivators and barriers for studying podiatry in Australia and New Zealand: A mixed methods study
Source: J Foot Ankle Res. 2024 Sep 4;17(3):e70004. doi: 10.1002/jfa2.70004 (PMC11372464; doi:10.1002/jfa2.70004)
Supplement: Supplementary file 1 — Supporting Information S1 [file JFA2-17-e70004-s003.pdf]

Motivators and barriers for studying podiatry in Australia and New Zealand: a mixed methods study

Michelle R Kaminski, Glen A Whittaker, Caroline Robinson, Matthew Cotchett, Malia Ho, Shannon E Munteanu, Mollie Dollinger, Sia Kazantzis, Xia Li, Ryan S Causby, Mike Frecklington, Steven Walmsley, Vivienne Chuter, Sarah L Casey, Burke Hugo, Daniel R Bonanno

---

Additional File 1. Good Reporting of A Mixed Methods Study (GRAMMS) checklist

GRAMMS checklist [1]

**ITEM**	**PAGE(S)**
1. Describe the justification for using a mixed methods approach to the research question	10
2. Describe the design in terms of purpose, priority and sequence of methods.	14-15
3. Describe each methods in terms of sampling, data collection and analysis.	10-15
4. Describe where integration has occurred, how it has occurred and who has participated in it.	15
5. Describe any limitation of one method associated with the presence of the other method.	N/A
6. Describe any insights gained from mixing or integrating methods.	35

1. O'Cathain A, Murphy E, Nicholl J. The quality of mixed methods studies in health services research. J Health Serv Res Policy. 2008;13(2):92-8.
