# Supplementary material for: Motivators and barriers for studying podiatry in Australia and New Zealand: A mixed methods study
Source: J Foot Ankle Res. 2024 Sep 4;17(3):e70004. doi: 10.1002/jfa2.70004 (PMC11372464; doi:10.1002/jfa2.70004)
Supplement: Supplementary file 3 — Supporting Information S3 [file JFA2-17-e70004-s001.pdf]

Motivators and barriers for studying podiatry in Australia and New Zealand: a mixed methods study

Michelle R Kaminski, Glen A Whittaker, Caroline Robinson, Matthew Cotchett, Malia Ho, Shannon E Munteanu, Mollie Dollinger, Sia Kazantzis, Xia Li, Ryan S Causby, Mike Frecklington, Steven Walmsley, Vivienne Chuter, Sarah L Casey, Burke Hugo, Daniel R Bonanno

---

Additional File 3. Non-podiatry student survey

Consent

Consent Form - Declaration by Participant

I (the participant) have read and understood the Participant Information Statement, and any questions have been answered to my satisfaction. I agree to participate in the study, I know I can withdraw at any time until [four weeks] following the collection of my data. I agree information provided by me or with my permission during the project may be included in a thesis, presentation and published in journals or reports on the condition that I cannot be identified. I consent for the use of non-identifiable data in future closely related research projects.

SECTION 1: DEMOGRAPHIC INFORMATION

Q1. What is your age?

____

Q2. What is your gender identity?

- ◯ Male
- ◯ Female
- ◯ Transgender
- ◯ Non-binary/non-conforming
- ◯ Prefer not to answer

Q3. What is your postcode?

____

Q4. What is your marital status?

- ◯ Never married
- ◯ Married
- ◯ De facto relationship
- ◯ Separated
- ◯ Divorced
- ◯ Widowed
- ◯ Prefer not to answer
- ◯ Other (please specify)

Please specify

____

Q5. Do you have any carer responsibilities (i.e. not from paid work)?

- ◯ Yes
- ◯ No

Who do you provide care for? (choose all that apply)

- ☐ Children and young adults (aged under 25)
- ☐ Parent(s)
- ☐ Grandparent(s)
- ☐ Sibling(s)
- ☐ Other family members (e.g. aunt/uncle, cousin)
- ☐ Friend(s)
- ☐ Neighbour(s)
- ☐ Other (please specify)

Please specify

____

Q6. Do you have any prior educational qualifications?

- ◯ Yes
- ◯ No

Please specify your qualification(s) (choose all that apply)	☐ Certificate I<br>☐ Certificate II<br>☐ Certificate III<br>☐ Certificate IV<br>☐ Diploma<br>☐ Advanced Diploma, Associate Degree<br>☐ Bachelor Degree<br>☐ Bachelor Honours Degree, Graduate Certificate, Graduate Diploma<br>☐ Masters Degree<br>☐ Doctoral Degree<br>☐ Other (please specify)
Please specify the other course(s)	__________________________________
Please specify the course(s)	__________________________________
Q7. What health/sport course are you currently studying?	◯ Physiotherapy<br>◯ Sport and exercise science<br>◯ Occupational therapy<br>◯ Speech pathology<br>◯ Dietetics/nutrition<br>◯ Orthoptics<br>◯ Prosthetics and orthotics<br>◯ Science (e.g. Health Science, Biomedicine)<br>◯ Other (please specify)
Please specify	__________________________________
Q8. Where are you currently studying your health/sport course?	◯ Auckland University of Technology<br>◯ Central Queensland University<br>◯ Charles Sturt University<br>◯ La Trobe University<br>◯ Southern Cross University<br>◯ The University of Newcastle<br>◯ University of South Australia<br>◯ University of Western Australia<br>◯ Western Sydney University
Q9. Are you enrolled in your course part-time or full-time?	◯ Part-time (i.e. completing 1 to 2 subjects or about 30 credit points or less)<br>◯ Full-time
What year are you currently completing (i.e. in what year are most of your subjects)?	◯ First year<br>◯ Second year<br>◯ Third year<br>◯ Fourth year
Q10. Are you an international student?	◯ Yes<br>◯ No
What is your home country?	__________________________________

SECTION 2: CHOOSING TO STUDY YOUR HEALTH/SPORT COURSE

Q11. What were you primarily doing the year prior to commencing your health/sport course?

- ◯ Final year of high school
- ◯ Studying another course
- ◯ Working
- ◯ Undertaking a gap year
- ◯ Other (please specify)

Please specify

____

Please specify the name of the course you were studying (e.g. Bachelor of Health Sciences)

____

What industry were you working in?

- ☐ Self-employed
- ☐ Health Care and Social Assistance
- ☐ Education and Training
- ☐ Accommodation and Food Services
- ☐ Retail Trade
- ☐ Administrative and Support Services
- ☐ Agriculture, Forestry, Fishing
- ☐ Mining
- ☐ Manufacturing
- ☐ Construction
- ☐ Electricity, Gas, Water, Waste Services
- ☐ Wholesale Trade
- ☐ Transport, Postal and Warehousing
- ☐ Information Media and Telecommunications
- ☐ Financial and Insurance Services
- ☐ Rental, Hiring and Real Estate Services
- ☐ Professional, Scientific, Technical Services
- ☐ Public Administration and Safety
- ☐ Arts and Recreation Services
- ☐ Other (please specify)

Please specify

____

Q12. How did you first hear about your health/sport profession? (choose all that apply)

- ☐ Work experience
- ☐ Career counsellor
- ☐ School teacher
- ☐ Family member
- ☐ Friend
- ☐ A health professional working in your health/sport profession
- ☐ A different health professional (i.e. someone not working in your health/sport profession)
- ☐ Social media
- ☐ University open day
- ☐ Career exhibitions and roadshows
- ☐ Association website(s) for your health/sport profession
- ☐ Other (please specify)

Please specify

____

Q13. To what extent did the following factors spark your interest in studying your health/sport course (i.e. to what extent did these factors motivate you)?

	Not applicable	Not at all	To a small extent	To a moderate extent	To a great extent
Interest in a health-related career	◯	◯	◯	◯	◯
Interest in a sport-related career	◯	◯	◯	◯	◯
Wanted to make a difference to peoples' health	◯	◯	◯	◯	◯
Opportunity to care for people from different backgrounds and age groups	◯	◯	◯	◯	◯
Inspired by a health professional	◯	◯	◯	◯	◯
Encouraged by a peer	◯	◯	◯	◯	◯
Encouraged by a family member	◯	◯	◯	◯	◯
Earning potential	◯	◯	◯	◯	◯
Could not get into another course	◯	◯	◯	◯	◯
Availability of scholarships and financial assistance	◯	◯	◯	◯	◯
Multiple career options post-graduation	◯	◯	◯	◯	◯
Flexible working hours	◯	◯	◯	◯	◯

Q14. When considering your future career path, to what extent did the following people influence your choice to study your health/sport course?

	Not applicable	Not at all	To a small extent	To a moderate extent	To a great extent
Myself	◯	◯	◯	◯	◯
Parent	◯	◯	◯	◯	◯
Family member	◯	◯	◯	◯	◯
Spouse / Partner	◯	◯	◯	◯	◯
Friend	◯	◯	◯	◯	◯
Career counsellor	◯	◯	◯	◯	◯
School teacher	◯	◯	◯	◯	◯
Health professional	◯	◯	◯	◯	◯
A health/sport student	◯	◯	◯	◯	◯
A recent health/sport graduate (within 3 years)	◯	◯	◯	◯	◯
Sporting coach	◯	◯	◯	◯	◯

Q15. In your opinion, to what extent do you think the following factors make your health/sport course an attractive profession?

	Not at all	To a small extent	To a moderate extent	To a great extent
Job prospects after graduation	◯	◯	◯	◯
Wide scope of practice	◯	◯	◯	◯
Ability to be involved in different areas of the profession (e.g. clinical, teaching, research)	◯	◯	◯	◯
Prospect of owning your own business	◯	◯	◯	◯
Offers pathways into other health or science disciplines (e.g. podiatry)	◯	◯	◯	◯
Ability to work in hospitals	◯	◯	◯	◯
Ability to work in community health	◯	◯	◯	◯
Ability to work in private practice	◯	◯	◯	◯
International employment opportunities	◯	◯	◯	◯

Q16. In your opinion, to what extent is your health/sport course a rewarding career choice?

0 - Not rewarding — 100 - Very rewarding

(Place a mark on the scale above)

Q17. Right now, how likely are you to recommend your health/sport course as a career?

◯ Extremely unlikely
◯ Unlikely
◯ Likely
◯ Extremely likely

Q18. Was your current health/sport course your first preference when applying to study?

◯ Yes
◯ No

What were your second and third choices?

____

What were the reason(s) why your current health/sport course was not your first preference?

____

When you applied for university, what courses were your top 3 preferences (list in order)?

____

Q19. Prior to your enrolment in your current health/sport course, did you ever consider studying podiatry?

◯ Yes
◯ No

To what extent did the following factors influence your consideration in studying podiatry?

	Not applicable	Not at all	To a small extent	To a moderate extent	To a great extent
Interest in a health-related career	◯	◯	◯	◯	◯
Wanted to make a difference to peoples' health	◯	◯	◯	◯	◯
Opportunity to care for people from different backgrounds and age groups	◯	◯	◯	◯	◯
Job prospects after graduation	◯	◯	◯	◯	◯
Flexible working hours	◯	◯	◯	◯	◯
Prospect of owning your own business	◯	◯	◯	◯	◯
Earning potential	◯	◯	◯	◯	◯
Availability of scholarships and financial assistance	◯	◯	◯	◯	◯
Wide scope of practice	◯	◯	◯	◯	◯
Ability to be involved in different areas of the profession (e.g. clinical, teaching, research)	◯	◯	◯	◯	◯
Offers pathways into other health or science disciplines (e.g. physiotherapy)	◯	◯	◯	◯	◯
Pathways for endorsement of scheduled medicines	◯	◯	◯	◯	◯
Pathways to become a podiatric surgeon	◯	◯	◯	◯	◯
Ability to work in varied clinical environments	◯	◯	◯	◯	◯
International employment opportunities	◯	◯	◯	◯	◯

When considering podiatry as a career, did you encounter any barriers or was there anything that deterred you from choosing to study podiatry?

◯ Yes
◯ No

What were the barriers (or deterrents) that you encountered (i.e. that may have influenced your decision not to study podiatry)?

____

Q20. Throughout your studies in your health/sport course, have you ever thought about leaving the course?

◯ Yes
◯ No

What were the reason(s) that made you think about leaving the course (i.e. what were the issues/concerns)? (choose all that apply)	☐ Family commitments<br>☐ Work commitments<br>☐ Financial hardship<br>☐ Health or stress<br>☐ Study/life balance<br>☐ Difficulties relating to workload<br>☐ Personal reasons<br>☐ Not enjoying the course<br>☐ Course was not as expected<br>☐ Change in mind regarding career path<br>☐ Other (please specify)
Please specify	__________________________________
Q21. If you weren't enrolled in your current health/sport course or you decided to leave the profession, what other courses or careers would you consider?	☐ Self-employed<br>☐ Health Care and Social Assistance<br>☐ Education and Training<br>☐ Accommodation and Food Services<br>☐ Retail Trade<br>☐ Administrative and Support Services<br>☐ Agriculture, Forestry, Fishing<br>☐ Mining<br>☐ Manufacturing<br>☐ Construction<br>☐ Electricity, Gas, Water, Waste Services<br>☐ Wholesale Trade<br>☐ Transport, Postal and Warehousing<br>☐ Information Media and Telecommunications<br>☐ Financial and Insurance Services<br>☐ Rental, Hiring and Real Estate Services<br>☐ Professional, Scientific, Technical Services<br>☐ Public Administration and Safety<br>☐ Arts and Recreation Services<br>☐ Other (please specify)
Please specify	__________________________________
Q22. If you weren't enrolled in your current health/sport course or you decided to leave the profession, would you consider studying podiatry?	◯ Yes<br>◯ No
Are there any specific reasons why you would not consider studying podiatry?	__________________________________

SECTION 3: KNOWLEDGE AND PERCEPTIONS OF THE PODIATRY PROFESSION

Q23. To the best of your knowledge, podiatrists can work within the following settings (choose all that apply):

- ☐ Acute/sub-acute hospitals
- ☐ Community health centres
- ☐ Private practice
- ☐ Aged care facilities
- ☐ Research/academia
- ☐ Teaching/clinical supervision

Q24. To the best of your knowledge, podiatrists are involved in the following specialty areas (choose all that apply):

- ☐ Sports medicine and rehabilitation
- ☐ Paediatrics
- ☐ High-risk foot (e.g. wound care, amputation prevention)
- ☐ Podiatric surgery (e.g. minor surgical procedures of the skin and nails)
- ☐ Geriatrics (i.e. providing care to elderly people)
- ☐ General podiatry (e.g. nail and skin care)

Q25. Compared to other allied health courses (e.g. physiotherapy, exercise science), how well do you think podiatry courses are promoted?

0 - Not well — 100 - Very well

(Place a mark on the scale above)

Q26. Compared to other allied health courses (e.g. physiotherapy, exercise science), how well do you think career opportunities in podiatry are promoted?

0 - Not well — 100 - Very well

(Place a mark on the scale above)

Q27. Select the social media platforms you use the most?

- ☐ Instagram
- ☐ Facebook
- ☐ Twitter
- ☐ TikTok
- ☐ Snapchat
- ☐ LinkedIn
- ☐ Other (please specify)

Please specify

____

Q28. To what extent do you think the following social media platforms would be appropriate and/or effective to source information about health/sport careers and the courses available?

	Not at all	To a small extent	To a moderate extent	To a great extent
Instagram	○	○	○	○
Facebook	○	○	○	○
Twitter	○	○	○	○
TikTok	○	○	○	○
Snapchat	○	○	○	○
LinkedIn	○	○	○	○

Q29. To what extent do you think the following advertising platforms would be effective for the promotion of health/sport careers and the courses available?

	Not at all	To a small extent	To a moderate extent	To a great extent
University websites	○	○	○	○
Association website(s)	○	○	○	○
Career exhibitions and roadshows	○	○	○	○
Social media (e.g. Instagram, Facebook, Twitter)	○	○	○	○
Career talks in high schools	○	○	○	○
Multi-media (e.g. advertisements in TV, radio, newspapers, magazines)	○	○	○	○

Q30. Do you have any final comments/ideas regarding your choice to study your health/sport course? Or how we may be able to attract more students to study podiatry? ______________________________
