# Supplementary material for: Motivators and barriers for studying podiatry in Australia and New Zealand: A mixed methods study
Source: J Foot Ankle Res. 2024 Sep 4;17(3):e70004. doi: 10.1002/jfa2.70004 (PMC11372464; doi:10.1002/jfa2.70004)
Supplement: Supplementary file 4 — Supporting Information S4 [file JFA2-17-e70004-s004.pdf]

Motivators and barriers for studying podiatry in Australia and New Zealand: a mixed methods study

Michelle R Kaminski, Glen A Whittaker, Caroline Robinson, Matthew Cotchett, Malia Ho, Shannon E Munteanu, Mollie Dollinger, Sia Kazantzis, Xia Li, Ryan S Causby, Mike Frecklington, Steven Walmsley, Vivienne Chuter, Sarah L Casey, Burke Hugo, Daniel R Bonanno

Additional File 4. Detailed participant characteristics

	**Podiatry (*n* = 278)**	**Non-podiatry (*n* = 553)**
Age*		
Mean (SD)	24.9 (8.48)	24.8 (8.17)
Median [Min, Max]	22.0 [17.0, 61.0]	21.0 [17.0, 62.0]
Gender identity, *n (%)*		
Male	92 (33.1)	130 (23.5)
Female	181 (65.1)	417 (75.4)
Transgender	0	3 (0.5)
Non-binary/non-conforming	3 (1.1)	3 (0.5)
Prefer not to answer	2 (0.7)	0
Marital status, *n (%)*		
Never married	207 (74.5)	400 (72.3)
Married	26 (9.4)	68 (12.3)
De facto relationship	25 (9.0)	56 (10.1)
Separated	2 (0.7)	5 (0.9)
Divorced	4 (1.4)	10 (1.8)
Widowed	6 (2.2)	7 (1.3)
Prefer not to answer	7 (2.5)	7 (1.3)
Other	1 (0.4)	0
Carer responsibilities, *n (%)*	37 (13.3)	91 (16.5)
Children and young adults (aged under 25)	15 (5.4)	73 (13.2)
Parent(s)	14 (5.0)	17 (3.1)
Grandparent(s)	1 (0.4)	2 (0.4)
Sibling(s)	7 (2.5)	10 (1.8)
Other family members (e.g. aunt/uncle, cousin)	6 (2.2)	6 (1.1)
Friend(s)	1 (0.4)	0
Neighbour(s)	0	0

	Podiatry (*n* = 278)	Non-podiatry (*n* = 553)
Other	5 (1.8)	3 (0.5)
Prior educational qualifications, *n (%)**	127 (45.7)	218 (39.4)
Certificate I	4 (1.4)	6 (1.1)
Certificate II	3 (1.1)	15 (2.7)
Certificate III	35 (12.6)	79 (14.3)
Certificate IV	22 (7.9)	57 (10.3)
Diploma	19 (6.8)	78 (14.1)
Advanced diploma, Associate Degree	2 (0.7)	11 (2.0)
Bachelor Degree	60 (21.6)	76 (13.7)
Bachelor Honours Degree, Graduate Certificate, Graduate Diploma	8 (2.9)	14 (2.5)
Masters Degree	9 (3.2)	9 (1.6)
Doctoral Degree	1 (0.4)	2 (0.4)
Other	6 (2.2)	6 (1.1)
University, *n (%)**		
Auckland University of Technology	15 (5.4)	88 (15.9)
Central Queensland University	11 (4.0)	45 (8.1)
Charles Sturt University	19 (6.8)	126 (22.8)
La Trobe University	48 (17.3)	66 (11.9)
Southern Cross University	8 (2.9)	0
The University of Newcastle	40 (14.4)	62 (11.2)
University of South Australia	75 (27.0)	90 (16.3)
University of Western Australia	19 (6.8)	8 (1.4)
Western Sydney University	43 (15.5)	67 (12.1)
Program of study, *n (%)*		
Physiotherapy		178 (32.2)
Sport and exercise science		35 (6.3)
Occupational therapy		161 (29.1)
Speech pathology		6 (1.1)
Dietetics / nutrition		37 (6.7)
Orthoptics		26 (4.7)
Prosthetics and orthotics		39 (7.1)
Science (e.g. health science, biomedicine)		71 (12.8)
Study load, *n (%)*		
Part-time†	21 (7.6)	78 (14.1)
Full-time	257 (92.4)	475 (85.9)
Year of study, *n (%)**		
First	76 (27.3)	152 (27.5)
Second	68 (24.5)	152 (27.5)
Third	81 (29.1)	152 (27.5)

	Podiatry (n = 278)	Non-podiatry (n = 553)
Fourth	53 (19.1)	96 (17.4)
International student, *n (%)*	17 (6.1)	19 (3.4)
Primary role in year prior to commencing program of study, *n (%)*[*]		
Final year of high school	109 (39.2)	212 (38.3)
Studying another course	62 (22.3)	106 (19.2)
Working	71 (25.5)	177 (32.0)
Self-employed	9 (3.2)	17 (3.1)
Health care and social assistance	34 (12.2)	67 (12.1)
Education and training	2 (0.7)	13 (2.4)
Accommodation and food services	6 (2.2)	9 (1.6)
Retail trade	13 (4.7)	22 (4.0)
Administrative and support services	0	16 (2.9)
Agriculture, forestry, fishing	1 (0.4)	1 (0.2)
Mining	0	2 (0.4)
Manufacturing	1 (0.4)	0
Construction	0	8 (1.4)
Electricity, gas, water, waste services	1 (0.4)	2 (0.4)
Wholesale trade	0	0
Transport, postal and warehousing	0	4 (0.7)
Information media and telecommunications	2 (0.7)	2 (0.4)
Financial and insurance services	1 (0.4)	7 (1.3)
Rental, hiring and real estate services	0	1 (0.2)
Professional, scientific, technical services	2 (0.7)	1 (0.2)
Public administration and safety	0	4 (0.7)
Arts and recreation services	2 (0.7)	11 (2.0)
Other	5 (1.8)	20 (3.6)
Undertaking a 'gap year'	24 (8.6)	35 (6.3)
Other	6 (2.2)	9 (1.6)
How did you first hear about your profession?		
Work experience	29 (10.4)	100 (18.1)
Career counsellor	17 (6.1)	63 (11.4)
School teacher	12 (4.3)	51 (9.2)
Family member	91 (32.7)	142 (25.7)
Friend	45 (16.2)	91 (16.5)
Health professional in profession	83 (29.9)	159 (28.8)
Other health professional	38 (13.7)	46 (8.3)
Social media	18 (6.5)	38 (6.9)
University open day	36 (12.9)	64 (11.6)
Career exhibitions and roadshows	13 (4.7)	32 (5.8)
Association websites	5 (1.8)	50 (9.0)

	**Podiatry (*n* = 278)**	**Non-podiatry (*n* = 553)**
Other	33 (11.9)	70 (12.7)

Data are n (%), unless otherwise specified.

*Maximum missing data were for 'Primary role in year prior to commencing program of study' (podiatry, *n* = 6; non-podiatry, *n* = 14). Missing data were for 'Age' (podiatry, *n* = 1), 'Prior educational qualifications' (podiatry, *n* = 1), 'University' (non-podiatry, *n* = 1), 'Year of study' (non-podiatry, *n* = 1), 'International student' (non-podiatry, *n* = 1).

†Part-time study load was defined as completing one to two subjects or 30 credit points or less.
